# A Conference on Hospital Diet

**Published:** 1910-06-25

**Authors:** Chas. E. Hecht

**Affiliations:** Secretary. 178 St. Stephen's House, Victoria Embankment, Westminster, S.W.


*To the Editor of* The Hospital.

### A CONFERENCE ON HOSPITAL DIET.

*To the Editor of* The Hospital.

Sir,—The Committee of the National Food Reform Association contemplate calling a conference at an early date to discuss the feeding of nurses in hospitals and other institutions. In the arrangements for such a meeting they are naturally anxious to secure the counsel and co-operation of some of the leading hospital and Poor Law matrons, from whom, as well as from any others interested, they would be glad to hear.

They would be much obliged if you would kindly give publicity to their intention in your columns.

Yours, etc., Chas. E. Hecht, Secretary.
178 St. Stephen's House,
Victoria Embankment,
Westminster, S.W., June 21, 1910.

